# Susceptibility-Weighted Imaging in Heat Stroke

**DOI:** 10.1371/journal.pone.0105247

**Published:** 2014-08-19

**Authors:** Xue-yan Zhang, Jun Li

**Affiliations:** 1 Nursing college, Binzhou Medical University, Yantai, Shandong, China; 2 Department of Radiology, Yantai Affiliated Hospital of Binzhou Medical University, Yantai, Shandong, China; University Medical Center (UMC) Utrecht, Netherlands

## Abstract

**Objective:**

To assess the role of susceptibility-weighted imaging in the detection of intracranial hemorrhage after heat stroke and in the prognosis.

**Materials and Methods:**

The study group consisted of eight patients after heat stroke, with a score of 3 to 9 in Glasgow Coma Scale. The MR studies were performed with a 1.5 T scanner. Susceptibility-weighted imaging data were collected within 2–5 days after heat stroke. The study was approved by ethics committee, and written informed consents were obtained from family members of the patients.

**Results:**

Punctate hemorrhages were detected in brain stem, corona radiata and frontal lobe by susceptibility-weighted imaging for three patients. Among the three cases, two patients came to death in the 5th day and the 25th day after heat stroke respectively. Another patient became a persistent vegetative state and died about 3 months later. Five patients with no hemorrhage detected gradually recovered and cerebellar dysfunction remained to various degrees.

**Conclusions:**

Heat stroke is a life-threatening condition characterized by hyperthermia and accompanied by various complications such as disseminated intravascular coagulation. Susceptibility-weighted imaging is a very useful tool for detection of intracranial hemorrhage and may probably evaluate the prognosis after heat stroke.

## Introduction

Heat stroke is characterized by an elevated core body temperature over 40°C and neurologic abnormalities including delirium, seizures, or coma [1], [2]. Potential immediate complications of severe heat stroke include shock, acute respiratory distress syndrome, acid-base or electrolyte disturbances, disseminated intravascular coagulation and rhabdomyolysis [1].

Heat stroke is generally reported in case reports or small patients' series. MRI findings [3] include hyperintense lesions in the cerebellum, midbrain, thalamus, hippocampus, splenium of corpus callosum, et al. Notably, heat-stroke shows the propensity to produce remarkable symmetric lesions in specific brain regions.

Disseminated intravascular coagulation syndrome may result in hemorrhagic lesions. Susceptibility-weighted imaging plays an important role in detecting intracranial hemorrhage [4]. Up to now, only case report on susceptibility-weighted imaging in heat stroke has been found [5]. Thus, the purpose of the present study is to evaluate the role of susceptibility-weighted imaging in the detection of intracranial hemorrhage after heat stroke and its prognosis.

## Materials and Methods

This study was approved by Ethic Committee of Binzhou Medical University. Family members of the patients provided their written informed consent to participate in this study. This consent procedure was approved by Ethics Committee of Binzhou Medical University. The study group included eight patients (5 men, 3 women) after heat stroke, with a score of 3 to 9 in Glasgow Coma Scale. Their average age was 68 years (range 43–90). All patients were admitted to the ICU with coma and fever (over 40°C) on hot days in August. Besides, one patient experienced generalized seizures. Complications including rhabdomyolysis, acute renal failure, acid-base or electrolyte disturbances and disseminated intravascular coagulation were diagnosed in five patients through laboratory examinations. Table 1 shows the platelet count, blood coagulation analysis and blood urine index of all patients collected within 1-2 days after admission.

**Table 1:** Statistical data of laboratory examinations for 8 patients with heat stroke.

Laboratory examinations	Death cases			Survival cases				
	1	2	3	1	2	3	4	5
Platelet count	↓	↓	↓	N	↓	N	↓	N
D2 dimer	↑	↑	↑	↑	↑	↑	↑	↑
Plasma prothrombin time	↑	↑	↑	N	N	N	↑	N
Prothrombin activity	↓	↓	↓	N	N	N	↓	N
International normalized ratio	↑	↑	N	N	N	N	↑	N
Activated partial thromboplastin time	↑	N	↑	N	N	N	N	N
Thromboplastin time	↑	N	↑	N	N	N	N	N
Plasma fibrinogen	↓	↓	↓	N	N	N	↓	N
Antithrombin III	↓	↓	↓	N	↓	N	↓	N
Urine red blood cell count	↑	↑	↑	N	↑	N	↑	↑
Urine occult blood	4+	1+	3+	2+	3+	-	1+	1+

N = normal.

The MR studies were performed by a SIEMENS Avanto 1.5 T magnetic resonance scanner with a standard quadrature head coil. The initial MR imaging including susceptibility-weighted imaging data were collected within 2–5 days after heat stroke. The MR imaging protocol included T1-weighted (TR450ms,TEl5ms), T2-weighted (TR3000ms, TE100ms), diffusion-weighted, FLAIR (TR8000ms, TE120ms) and susceptibility-weighted imaging. Imaging parameters of susceptibility-weighted imaging were as follows: TR 49ms, TE 40ms, slice thickness 2mm, 56 slices in a single slab.

All patients were divided into two groups according to whether hemorrhage was detected or not by susceptibility-weighted imaging. The number of deaths and survivals were counted respectively. Statistical analysis was performed by the Statistical Package for the Social Sciences (SPSS, version 17). Fisher's exact test was performed to compare the differences. The differences were considered statistically significant when P<0.05.

## Results

**Figure 1:**
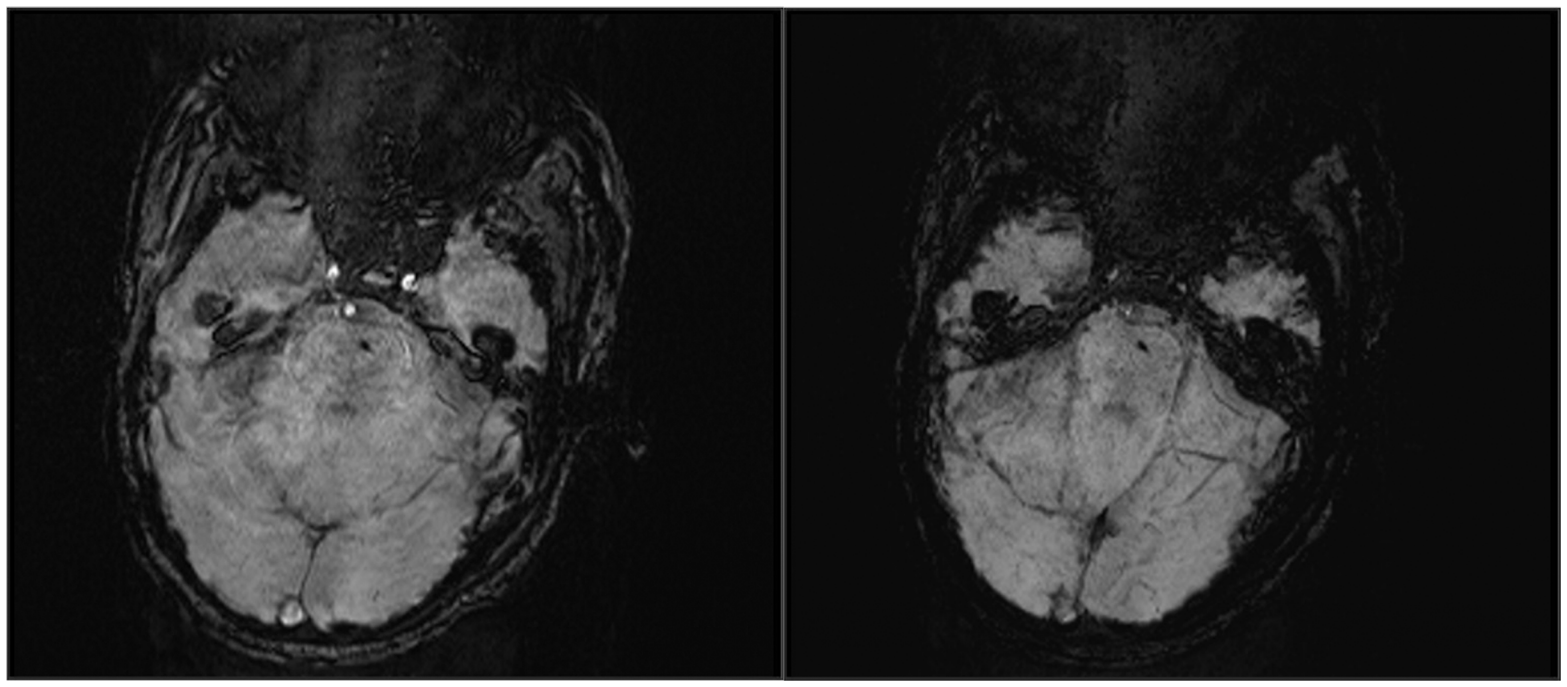
SWI obtained in a 64-year-old woman after heat stroke with a score of 3 in Glasgow Coma Scale. On the 5th day after admission, amplitude (A) and minIP (B) images show punctate hemorrhage detected in brain stem.

**Figure 2:**
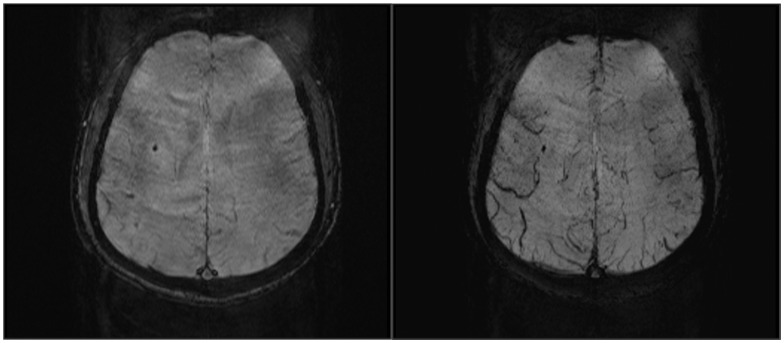
SWI obtained in a 43-year-old man after heat stroke with a score of 3 in Glasgow Coma Scale. On the 4th day after admission, amplitude (A) and minIP (B) images show punctate hemorrhage detected in right corona radiata.

**Figure 3:**
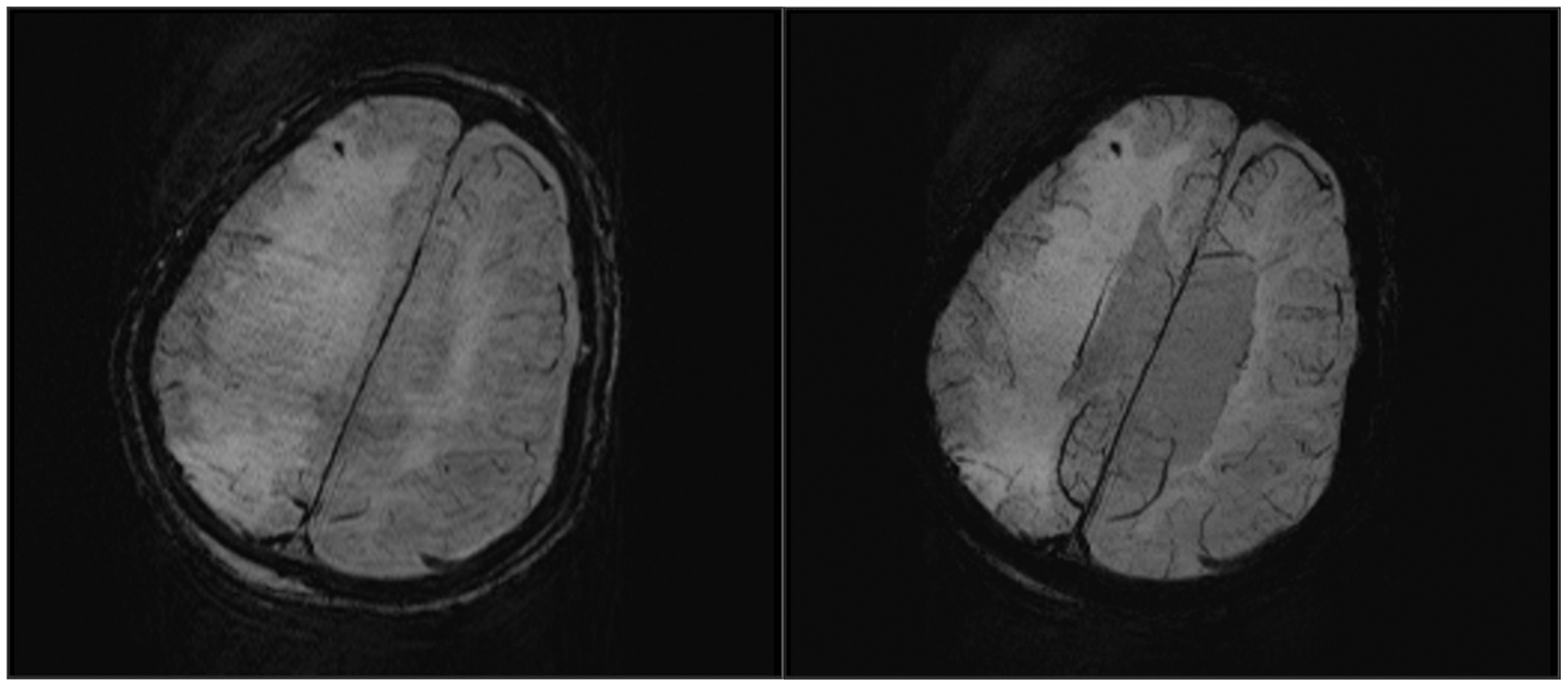
SWI obtained in a 90-year-old man after heat stroke with a score of 6 in Glasgow Coma Scale. On the 4th day after admission, amplitude (A) and minIP (B) images show punctate hemorrhage detected in right frontal lobe.

Routine MR sequences revealed some abnormal signals as follows. Diffusion-weighted imaging revealed symmetrical restricted water diffusion in the bilateral dentate nuclei for one patient. T2-weighted and FLAIR imaging revealed remarkable symmetric hyperintensity in the cerebellar peduncles for one patient. T2-weighted and FLAIR imaging revealed mild symmetric hyperintensity in the cerebellar hemispheres for one patient. Acute massive cerebral infarction of the right hemisphere occured in one patient. Acute lacunar infarct of the left centrum ovale occurred in one patient. In addition, hemorrhages were detected in three patients by susceptibility-weighted imaging. The description of the type and location of the hemorrhages were presented in Table 2. Punctate hemorrhage was detected in brain stem (Fig. 1) of one patient after heat stroke with a score of 3 in Glasgow Coma Scale. Similar punctate hemorrhage was detected in right corona radiata (Fig. 2) of one patient after heat stroke with a score of 3 in Glasgow Coma Scale too. For another patient after heat stroke with a score of 6 in Glasgow Coma Scale, and punctate hemorrhage was detected in right frontal lobe (Fig. 3).

**Table 2:** Description of the type and location of the hemorrhages detected by SWI.

Hemorrhage	Case 1	Case 2	Case 3
Type	Punctate	Punctate	Punctate
Location	Brain stem	Right corona radiata	Right frontal lobe

Among the three cases with hemorrhages detected, one patient died in the 5th day after heat stroke. One patient with persistent coma suffered from encephalitis in the 16th day and died in the25th day. Another patient became a persistent vegetative state and died about 3 months later. However, five patients with no hemorrhage detected gradually recovered and cerebellar dysfunction remained to various degrees. The number of deaths and survivals in two groups are shown in Table 3 based on that whether hemorrhage was detected or not. Case fatality rate had statistical differences in two groups (P = 0.018).

**Table 3:** Statistical data of 8 patients with heat stroke according to SWI and prognosis.

SWI	The number of deaths	The number of survivals
Hemorrhage	3	0
No hemorrhage	0	5

Fisher's exact test; p = 0.018.

## Discussion

Heat stroke is a life-threatening condition characterized by severe hyperthermia associated with central nervous system abnormalities (including delirium, seizures, or coma) [6] and ofen accompanied by various complications such as disseminated intravascular coagulation [1], [2], [7].

MRI findings of heat stroke include lesions in dentate nuclei [3], cerebellar hemispheres [5], [8], [9], cerebellar peduncles, midbrain, thalami [10], hippocampi [11], basal ganglia [7], the splenium [3], temporo-occipital lobes [12]. Studies have confirmed the selective vulnerability of cerebellar Purkinje cells to heat-induced injury [3], [13]. A cytotoxic and/or excitotoxic mechanism has been suggested [5], possibly of ischemic nature [3] resulting from hypoperfusion induced by vascular endothelial damage which usually accompanies heat-stroke [14]. Our results are in agreement with previous studies. In this study, symmetrical lesions were found in dentate nuclei, cerebellar hemispheres and cerebellar peduncles.

Murcia-Gubianas C et al. [4] detected hemorrhages in cerebellum after heat stroke by susceptibility-weighted imaging. Sonkar SK et al. [7] detected bilateral intracerebral hemorrhages in basal ganglia of one case. Unlike previous studies, punctate hemorrhages were detected in brain stem, corona radiata and frontal lobe by susceptibility-weighted imaging in this study. Table 1 shows the platelet count, blood coagulation analysis and blood urine index of all patients collected within 1–2 days after admission. The suggested explanation for intracerebral hemorrhage with heat stroke is concomitant complication of disseminated intravascular coagulation. In this study, all the patients with intracerebral hemorrhages detected by susceptibility-weighted imaging eventually died. Intracerebral hemorrhages induced by heat stroke may be an important index for bad outcome.

There are several limitations to this present study. Many patients after heat stroke admitted to the Intensive Care Unit were intubated and image quality was influenced. In addition, the sample size of this study was fairly small, further studies are needed to expand the sample size.

In summary, heat stroke is a life-threatening condition characterized by hyperthermia and accompanied by various complications such as disseminated intravascular coagulation. Susceptibility-weighted imaging is a very useful tool for detection of intracranial hemorrhage and may probably evaluate the prognosis after heat stroke.
